# Highly potent inhibitors of cathepsin K with a differently positioned cyanohydrazide warhead: structural analysis of binding mode to mature and zymogen-like enzymes

**DOI:** 10.1080/14756366.2021.2024527

**Published:** 2022-02-11

**Authors:** Jakub Benýšek, Michal Buša, Petra Rubešová, Jindřich Fanfrlík, Martin Lepšík, Jiří Brynda, Zuzana Matoušková, Ulrike Bartz, Martin Horn, Michael Gütschow, Michael Mareš

**Affiliations:** a Institute of Organic Chemistry and Biochemistry of the Czech Academy of Sciences, Prague, Czech Republic; b First Faculty of Medicine, Charles University, Prague, Czech Republic; c Department of Biochemistry, Faculty of Science, Charles University, Prague, Czech Republic; d Department of Natural Sciences, University of Applied Sciences Bonn-Rhein-Sieg, Rheinbach, Germany; e Pharmaceutical Institute, Pharmaceutical & Medicinal Chemistry, University of Bonn, Germany

**Keywords:** Cathepsin K, protease inhibitor, cyanohydrazide warhead, azadipeptide nitrile, structure

## Abstract

Cathepsin K (CatK) is a target for the treatment of osteoporosis, arthritis, and bone metastasis. Peptidomimetics with a cyanohydrazide warhead represent a new class of highly potent CatK inhibitors; however, their binding mechanism is unknown. We investigated two model cyanohydrazide inhibitors with differently positioned warheads: an azadipeptide nitrile **Gü1303** and a 3-cyano-3-aza-β-amino acid **Gü2602**. Crystal structures of their covalent complexes were determined with mature CatK as well as a zymogen-like activation intermediate of CatK. Binding mode analysis, together with quantum chemical calculations, revealed that the extraordinary picomolar potency of **Gü2602** is entropically favoured by its conformational flexibility at the nonprimed-primed subsites boundary. Furthermore, we demonstrated by live cell imaging that cyanohydrazides effectively target mature CatK in osteosarcoma cells. Cyanohydrazides also suppressed the maturation of CatK by inhibiting the autoactivation of the CatK zymogen. Our results provide structural insights for the rational design of cyanohydrazide inhibitors of CatK as potential drugs.

## 1. Introduction

**Figure 1.**
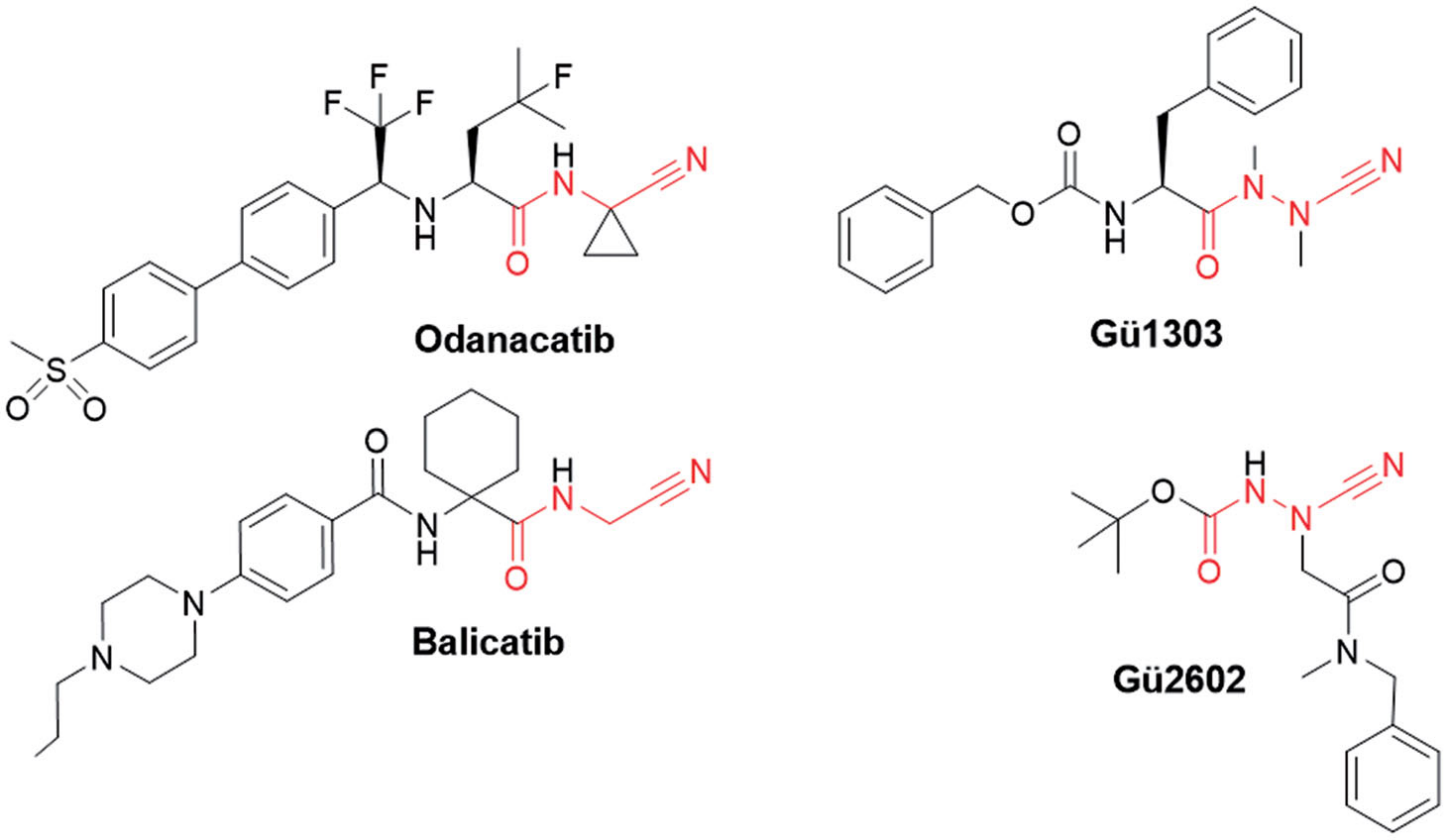
Chemical structures of peptidomimetic inhibitors of cathepsin K with a reactive nitrile functionality. The warhead is indicated in red. The dipeptide nitriles odanacatib and balicatib were developed as osteoporosis drugs. The azadipeptide nitrile **Gü1303** and 3-cyano-3-aza-β-amino acid **Gü2602** contain the cyanohydrazide warhead.

Cathepsin K (CatK) is one of the most investigated cysteine cathepsins, both by academia and pharmaceutical companies. It is expressed in high levels in osteoclasts, where it serves as the principal protease involved in bone remodelling. It has been validated as a therapeutic target for osteoporosis, an increasing health problem in the modern world^1–4^. This disorder is caused by progressive loss of bone mass due to excessive activity of osteoclastic CatK. Several anti-remodelling inhibitors of CatK, such as odanacatib or balicatib^5–7^ (Figure 1), have been developed for the treatment of osteoporosis but have not yet been approved. CatK has also been implicated in the pathophysiology of two common forms of arthritis, osteoarthritis and rheumatoid arthritis^8^^,^^9^. In bone and cartilage disorders, CatK functions as a potent collagen degrading enzyme with the unique ability to cleave the triple helix of collagen molecules at multiple locations, an activity that is unparalleled among human collagenases^10^^,^^11^. This activity is induced by glycosaminoglycans, foremost chondroitin-4-sulphate, which mediates the formation of a complex between CatK and the collagen substrate^10^^,^^12^^,^^13^.

Further, there is increasing evidence that CatK is a pro-tumorigenic protease that plays an important role in processes associated with tumour growth, invasion, and metastasis of cancer cells and their interactions with the tumour microenvironment (for review see^14^^,^^15^). Its complex action includes direct degradation of collagen and other extracellular matrix proteins, e.g. in bone metastases, and indirect affecting of the signalling pathways^16–19^. In glioma, CatK can regulate cancer stem-like cell mobilisation and proteolytically modulate levels of chemokines and growth factors^20^. Thus, clinical research is investigating CatK as a marker for diagnosis and survival prognosis in metastatic cancer and as a target for anticancer inhibitors^21^^,^^22^.

At the protein level, CatK activity is regulated by endogenous protein inhibitors^23^^,^^24^ and by zymogen activation^24–26^. CatK is synthesised as an inactive zymogen (procathepsin) in which the N-terminal propeptide blocks the active site^27^^,^^28^. Activation to the mature, active form occurs upon proteolytic removal of the N-terminal propeptide (also termed the “activation peptide”). This process was shown to be autocatalytic and bimolecular; it is triggered by acidic pH and also enhanced by interaction with chondroitin-4-sulfate^25^^,^^29^. The activation pathway of CatK includes an activation intermediate with a partially processed propeptide^25^, which has not been studied in detail so far, and its spatial structure remains unknown.

Peptidomimetics with a reactive nitrile functionality have attracted particular attention as potent inhibitors of CatK and other cysteine cathepsins. The electrophilic nitrile warhead allows for covalent interaction with the catalytic cysteine nucleophile, leading to the reversible formation of a covalent thioimidate adduct. Representatives of such CatK inhibitors are dipeptide nitriles odanacatib and balicatib^5–7^ (Figure 1). An exchange of the α-CH moiety of the P1 amino nitrile by a nitrogen atom led to azadipeptide nitriles with the cyanohydrazide warhead forming a stabilised isothiosemicarbazide adduct^30–32^. They have been introduced as a class of efficient covalent-reversible inhibitors of human cysteine cathepsins, including CatK, and their homologs from parasites and pathogens^30–34^. Compared to their parent carbapeptide analogs, bioactive azapeptides can possess improved potency, selectivity, and pharmacokinetics^35–38^. The 3-cyano-3-aza-β-amino acid derivatives represent another scaffold bearing the cyanohydrazide warhead^39^. They were designed to position the warhead centrally in the peptidomimetic inhibitor molecule for extended interactions of inhibitor substructures with the non-primed and primed binding regions of the target enzymes. These compounds were found to be exceptionally potent, in particular towards CatK^39^.

The exact binding mode of peptidomimetics with the cyanohydrazide warhead to CatK had not been characterised so far. To do this, we investigated two model cyanohydrazide inhibitors selective for CatK with high potency in the subnanomolar to picomolar range, namely the azadipeptide nitrile **Gü1303**^30^^,^^31^ and the 3-cyano-3-aza-β-amino acid derivative **Gü2602**^39^ (Figure 1). The crystal structures of their complexes with mature CatK and the activation intermediate of CatK were determined and functional properties *in vitro* and in cells were described. The present structures and inhibitor interaction data provide a footing for the rational design of next generation cyanohydrazide inhibitors of CatK as potential therapeutics.

## 2. Materials and methods

### 2.1. Materials

Inhibitors **Gü1303** and **Gü2602** and the CatK activity-based probe **25** were synthesised as described previously^30^^,^^39^^,^^40^.

### 2.2. Expression and purification of the recombinant zymogen of cathepsin K

Human CatK (Uniprot accession number P43235) was expressed in the X-33 strain of the methylotrophic yeast *Pichia pastoris* (Thermo Fisher). A gene coding for the zymogen form of CatK was purchased from GenScript and recloned into expression plasmid pPICZαA (Thermo Fisher) using XhoI and NotI restriction sites. Transformation of *P. pastoris* cells and protein expression were carried out as described previously^41^^,^^42^. The yeast medium containing the recombinant CatK zymogen was centrifuged (2,500 g for 10 min), and the supernatant was lyophilised and dissolved in 20 mM MES pH 6.0 (to 10% of the original volume). The protein solution was then desalted over a Sephadex G-25 column equilibrated with the same buffer. The CatK zymogen was purified using chromatography on Mono S (HR 5/5 column) equilibrated with 50 mM sodium acetate pH 5.5, and eluted by a linear gradient of 2 M NaCl. The purified protein was concentrated to 2 mg/ml using an Amicon Ultracel-10k centrifugal filter device (Millipore).

### 2.3. Activation of the cathepsin K zymogen and preparation of inhibitor complexes

The purified CatK zymogen (75 µM) was activated by incubation in 0.1 M sodium acetate pH 4.0 containing 2.5 mM DTT, 1 mM EDTA, and 0.3 M NaCl under an argon atmosphere at room temperature. The zymogen-like activation intermediate iCatK was obtained after 30 min of incubation, and fully activated mature enzyme mCatK after 75 min. Activation was terminated by the addition of 6-fold molar excess of the inhibitor **Gü1303** or **Gü2602,** followed by incubation under argon atmosphere for 3 h at room temperature. The activation and inhibition were monitored with a kinetic activity assay using the fluorogenic substrate Cbz-Gly-Pro-Arg-AMC (Cbz, benzyloxycarbonyl; AMC, 7-amino-4-methylcoumarin) and Laemmli-SDS-PAGE. The processing sites were identified by N-terminal protein sequencing after electroblotting of Laemmli-SDS-PAGE gels to a PVDF membrane using a Procise 494 cLC protein sequencer (Applied Biosystems) and by peptide mapping using mass spectrometry (LC-MS/MS) on an LTQ Orbitrap XL mass spectrometer (Thermo Scientific) coupled to a UHPLC system. The LC–MS/MS data were processed with Bioworks software (Thermo). The complexes were buffer-exchanged into 20 mM sodium acetate pH 5.5 containing 2.5 mM DTT and 0.25 M NaCl, and concentrated to 3.5 mg/ml for mCatK and 5 mg/ml for iCatK using an Amicon Ultracel-10k centrifugal filter device; the inhibitors were maintained during buffer exchange and concentration in a 6-fold molar excess to mCatK/iCatK in the mixture.

### 2.4. Autoactivation assay with the cathepsin K zymogen and inhibitors

The CatK zymogen (2.8 µM) was incubated at room temperature in the presence or absence of the inhibitor (10 µM **Gü1303** or **Gü2602**) in 150 µL of 0.1 M sodium acetate pH 4.0 containing 2.5 mM DTT and 0.3 M NaCl for 0, 30 and 240 min. The reaction was terminated by the addition of E-64 (10 µM final concentration), followed by acetone precipitation; the reaction mixture was separated by Laemmli-SDS-PAGE.

### 2.5. Protein crystallisation and data collection

Crystals were obtained by the vapour diffusion technique in hanging drops at 18 °C. Drops consisted of 1 µl of the protein-inhibitor complex, 0.15 µl 1:100 diluted seed stock and 1 µl of the reservoir solution. The drops were equilibrated over the following reservoir solutions: (1) 10% PEG 8000, 20% ethylene glycol, 0.02 M sodium L-glutamate, 0.02 M DL-alanine, 0.02 M glycine, 0.02 M DL-lysine HCl, 0.02 M DL-serine, 0.1 M MES/imidazole pH 6.5 for iCatK-**Gü1303** complex; (2) 10% PEG 8000, 20% ethylene glycol, 0.02 M sodium formate, 0.02 M ammonium acetate, 0.02 M trisodium citrate, 0.02 M sodium potassium L-tartrate, 0.02 M sodium oxamate, 0.1 M MES/imidazole pH 6.5 for iCatK-**Gü2602** complex; (3) 12.5% PEG 1000, 12.5% PEG 3350, 12.5% MPD, 0.03 M sodium nitrate, 0.03 M disodium hydrogen phosphate, 0.03 M ammonium sulphate, 0.1 M MES/imidazole pH 6.5 for mCatK-**Gü1303** complex; and (4) 10% PEG 8000, 20% ethylene glycol, 0.02 M sodium formate, 0.02 M ammonium acetate, 0.02 M trisodium citrate, 0.02 M sodium potassium L-tartrate, 0.02 M sodium oxamate, 0.1 M MES/imidazole pH 6.5 for mCatK-**Gü2602** complex. Crystals were flash-cooled by plunging them into liquid nitrogen, and diffraction data from the crystals of mCatK complexes were collected at 100 K on a MicroMax-007 HF Microfocus rotating anode X-ray generator equipped with a PILATUS 300 K detector (Rigaku). Data from the crystals of iCatK complexes were collected at 100 K on an MX 14.1 beamline operated by Helmholtz-Zentrum Berlin at the BESSY II electron storage ring in Berlin-Adlershof, Germany^43^. All diffraction data was processed using the XDS suite of programs^44^. Crystal parameters, data collection statistics, and final refinement statistics are in Table S1.

### 2.6. Structure determination, refinement and analysis

The structures of the mCatK-**Gü1303/Gü2602** complexes and the main domain of iCatK in complex with **Gü2602** were solved by molecular replacement with the program MolRep^45^ from the CCP4 program suite^46^ using the structure of human cathepsin K (PDB code: 7NXM)^40^ as a search model. The propeptide domain structure in iCatK-**Gü2602** was built using the *de novo* model building program Buccaneer^47^. The structure of the iCatK-**Gü1303** complex was solved by molecular replacement using the structure of iCatK-**Gü2602** as a search model. Model refinement was carried out using the program REFMAC 5.5, interspersed with manual adjustments using Coot. The geometric restraints for ligands were constructed by the program AceDRG^48^. The quality of the final models was validated with MolProbity^49^. The final refinement statistics are given in Table S1. Atomic coordinates and structure factors have been deposited in the Protein Data Bank with the accession codes: 7QBL, 7QBN, 7QBM, and 7QBO for mCatK-**Gü2602**, mCatK-**Gü1303**, iCatK-**Gü2602,** and iCatK-**Gü1303**, respectively. Inhibitor interactions were analysed using the programs CONTACT^46^ and PLIP^50^. The distance cut-offs were set to 3.3 Å for hydrogen bonds and 4.2 Å for contacts. The nonpolar interactions represent contacts between two hydrophobic atoms defined as carbon atoms having carbon or hydrogen atoms as neighbours. All figures showing structural representations were prepared with the PyMOL Molecular Graphics System, version 1.40 (Schrödinger, LLC).

### 2.7. Molecular modelling

The X-ray structure of the mCatK-**Gü2602** complex was used for molecular modelling. Hydrogen atoms were added to the protein by the Reduce and Leap programs in AMBER 14^51^. The Asp, Glu, Lys, Arg, and His residues were charged, with the exception of Asp82 and His162. Hydrogen atoms of the inhibitor were added manually using the PyMOL. The ff14SB force field^51^ was used for the protein, while the GAFF force field^51^ and RESP charges at HF/6-31G* level were used for the ligand. The molecular dynamics/quenching (MD/Q) technique was used to search for possible conformations of **Gü2602** in the complex. The simulations were performed using AMBER 14. During the simulations, the *N*-benzyl-*N*-methylformamide segment of the inhibitor was relaxed; specifically, the C, O, and N heavy atoms in this segment and all H atoms of the inhibitor were relaxed while the rest of the system was frozen. The structures were collected every 1 ps in two independent runs with two orientations of the relaxed inhibitor segment (i.e. the heavy atoms as above). The simulations were 10 ps long at 600 K using 1 fs time step and a Berendsen thermostat. All the obtained structures were optimised (i.e. residues within 6 Å of the inhibitor were relaxed) by using the corrected semiempirical quantum mechanical (SQM) PM6-D3H4 method^52^^,^^53^. The environment was described by the COSMO implicit solvent model^54^^,^^55^. The SQM calculations were done by Cuby4^56^ and MOPAC2016^57^. Residues further than 6 Å from the inhibitor were frozen during the optimisation.

### 2.8. Cathepsin K activity and inhibition assays

Enzymatic activity of mCatK was measured using a kinetic continuous assay with the fluorogenic substrate Cbz-Gly-Pro-Arg-AMC (Bachem). The assay was performed in a 96-well microplate format in a total assay volume of 100 µl at 37 °C. The assay mixture contained an aliquot of mCatK (e.g. from the autoactivation assay) and 20 µM Cbz-Gly-Pro-Arg-AMC in 0.1 M sodium acetate pH 5.5 containing 2.5 mM DTT, 0.15 M NaCl, 0.1% PEG 6000, and 1 mM EDTA. The kinetics of the product release were continuously monitored in an Infinite M1000 microplate reader (Tecan) at excitation and emission wavelengths of 360 and 465 nm, respectively. The Michaelis-Menten kinetic parameters were determined by measuring the rate of hydrolysis of the substrate (0–100 μM) using the same assay with mCatK (0.4 nM); the *K*_m_ value obtained by nonlinear regression using GraFit software was 17.4 µM.

Inhibition measurements were performed analogously. mCatK (0.42 nM) was added to a mixture of the fluorogenic substrate Cbz-Gly-Pro-Arg-AMC (20 µM) and an inhibitor (0–100 nM) in 0.1 M sodium acetate pH 5.5 containing 2.5 mM DTT, 0.15 M NaCl, 0.1% PEG 6000, and 1 mM EDTA. The substrate hydrolysis was monitored for 40 min. For the slow-binding inhibitor **Gü1303**, an observed first-order rate constant *k*_obs_ was calculated at each inhibitor concentration by fitting the progress curve to the equation P = v_s_t + (v_i_ - v_s_)(1 - exp(*k*_obs_t))/*k*_obs_ + d, where P is the product formation, v_s_ is the steady-state velocity, t is the reaction time, v_i_ is the initial velocity, and d is offset. The apparent inhibition constant *K*_i_^′^ was determined by non-linear regression using equation v_s_/v_0_ = 1/(1 + [I]/*K*_i_^′^). The true inhibition constants *K*_i_ were calculated using the Cheng Prusoff equation *K*_i_ = *K*_i_^′^/(1 + [S]/*K*_m_), where [S] is the substrate concentration and *K*_m_ is the Michaelis constant. The apparent second-order rate constant *k*_on_^′^ was determined by fitting to the linear equation *k*_obs_ = *k*′_on_ [I] + *k*_off_, and the true constant *k*_on_ was calculated by correction *k*_on_ = *k*_on_^′^(1 + [S]/*K*_m_). The fast-binding inhibitor **Gü2602** showed linear progress curves, and the apparent inhibition constant *K*_i_^′^ was determined by non-linear regression using the Morrison equation for tight binding inhibition^58^ with GraphPad Prism software. The concentration of mCatK was determined by active site titration as described previously^59^ with E-64 used as the titrant^60^. The final concentration of DMSO in the assay systems did not exceed 1.5%.

### 2.9. Osteosarcoma cell imaging

Imaging of cathepsin K in human bone osteosarcoma cells (U-2 OS) was done as described previously using the CatK activity-based probe **25**^40^. U-2 OS cells were cultivated in McCoy’s 5 A medium supplemented with 10% FBS, 2 mM glutamax (L-alanyl-L-glutamine), 100 units/ml penicillin, and 100 μg/ml streptomycin at 37 °C in 5% CO_2_. Fifty thousand cells were seeded into a well of a 12-well plate and allowed to attach. After 24 h, 1 μM probe was added, and cells were incubated for 16 h. Cells were detached using 0.25% trypsin-EDTA solution ( Merck), collected by centrifugation (500 g for 5 min), washed with PBS, resuspended in 100 μl of loading buffer, and heated at 100 °C for 10 min. The competitive labelling was performed after the preincubation of cells with 1 μM **Gü1303** or **Gü2602** for 3 h. The samples (30 μl) were separated on 4–12% Bis-Tris polyacrylamide gels (Thermo Fisher Scientific). The gels were visualised using a Typhoon RGB imager (GE Healthcare Life Sciences) with excitation at 635 nm and emission at 660 nm (long pass filter).

## 3. Results

### 3.1. Cyanohydrazides are potent inhibitors of cathepsin K activity and zymogen activation both *in vitro* and in cells

**Figure 2.**
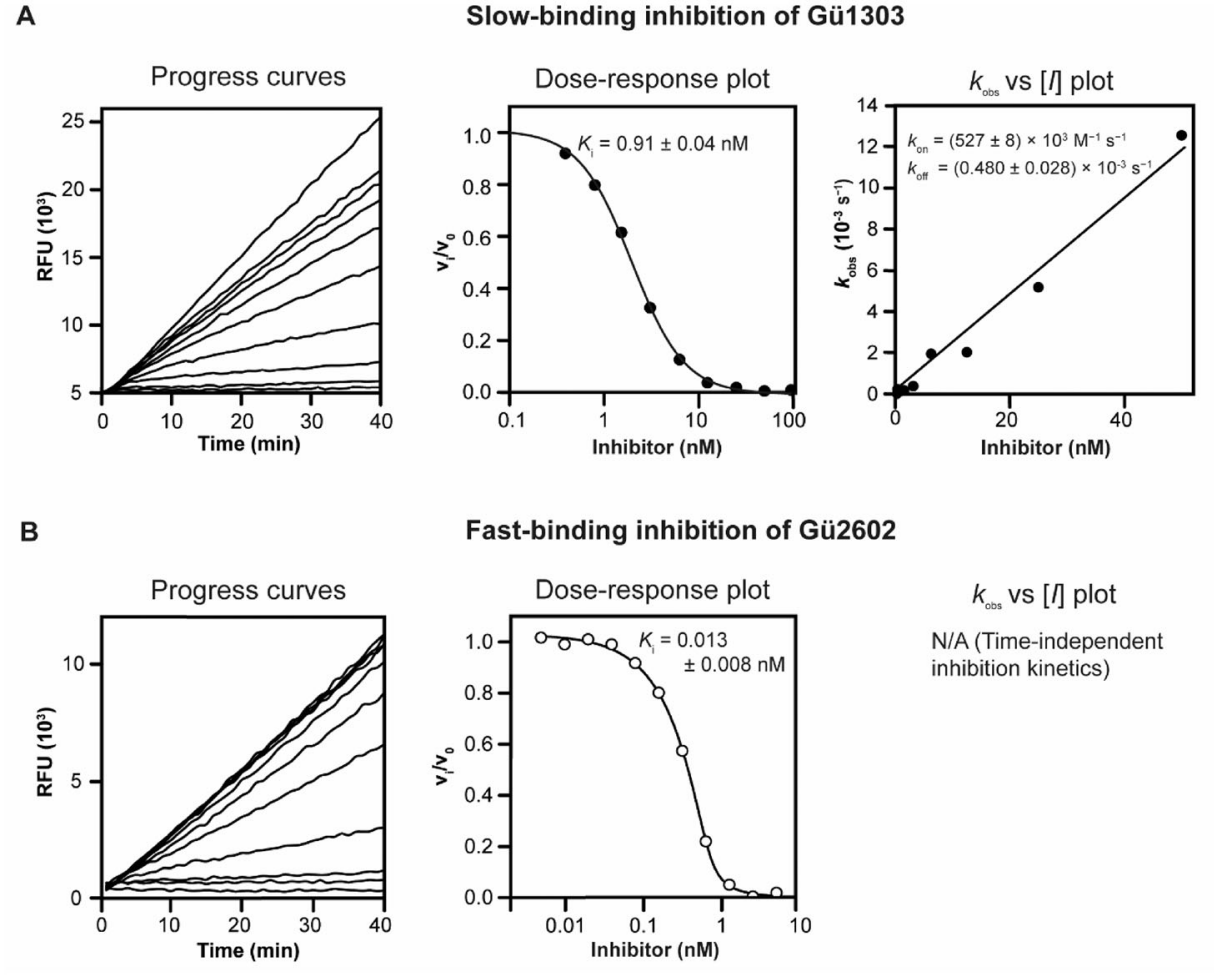
Different inhibition kinetics of mature cathepsin K with **Gü1303** and **Gü2602.** Progress curves show the hydrolysis of the fluorogenic substrate Cbz-Gly-Pro-Arg-AMC by mCatK at pH 5.5 in the presence of increasing inhibitor concentrations. (A) **Gü1303** exhibited a time-dependent inhibition characterised by non-linear progress curves typical of slow-binding kinetics. (B) Linear progress curves obtained for **Gü2602** are characteristic of fast-binding inhibitors. In dose–response plots, the derived steady-state reaction velocities were plotted against inhibitor concentration, and the inhibition constants *K*_i_ were obtained after correction by the Cheng-Prusoff and Morrison equations (see Materials and Methods). In the *k*_obs_ versus [*I*] plot, the first-order rate constants *k*_obs_ from the time-dependent progress curves were plotted against inhibitor concentrations to show a linear dependence.

Functional properties of the azadipeptide nitrile inhibitor **Gü1303**^30^ and the 3-cyano-3-aza-β-amino acid inhibitor **Gü2602**^39^ were characterised using an *in vitro* kinetic assay with the mature form of recombinant human cathepsin K (mCatK) and the fluorogenic substrate Cbz-Gly-Pro-Arg-AMC. Subnanomolar values of the inhibition constant *K*_i_ were determined: Notably, we found a *K*_i_ of 0.91 nM for **Gü1303** and an almost two orders of magnitude lower *K*_i_ of 0.013 nM for **Gü2602** (Table 1). A detailed analysis of the kinetic behaviour showed non-linear progress curves that indicated time-dependent inhibition for **Gü1303** typical for slow-binding inhibitors (Figure 2(A)). This allowed for the calculation of the second-order rate constant of inactivation *k*_on_ of 527 × 10^3^ M^−1^s^−1^, thus demonstrating the extraordinary potency of **Gü1303** (Table 1). In contrast, linear progress curves were obtained for **Gü2602** that are characteristic of fast-binding inhibitors (Figure 2(B)).

**Table 1.** Inhibition of human mature cathepsin K.

Compound	mCatK inhibition^a^		
	*K*_i_ (nM)	*k*_on_ (10^3^ M^−1^ s^−1^)	*k*_off_ (10^−3^ s^−1^)
**Gü2602**	0.013 ± 0.008	n.d.^b^	n.d.^b^
**Gü1303**	0.91 ± 0.04	527 ± 8	0.48 ± 0.03

^a^The inhibition parameters were measured using a kinetic activity assay with the fluorogenic peptide substrate Cbz-Gly-Pro-Arg-AMC at pH 5.5. ^b^n.d.: not determined for linear progress curves.

**Figure 3.**
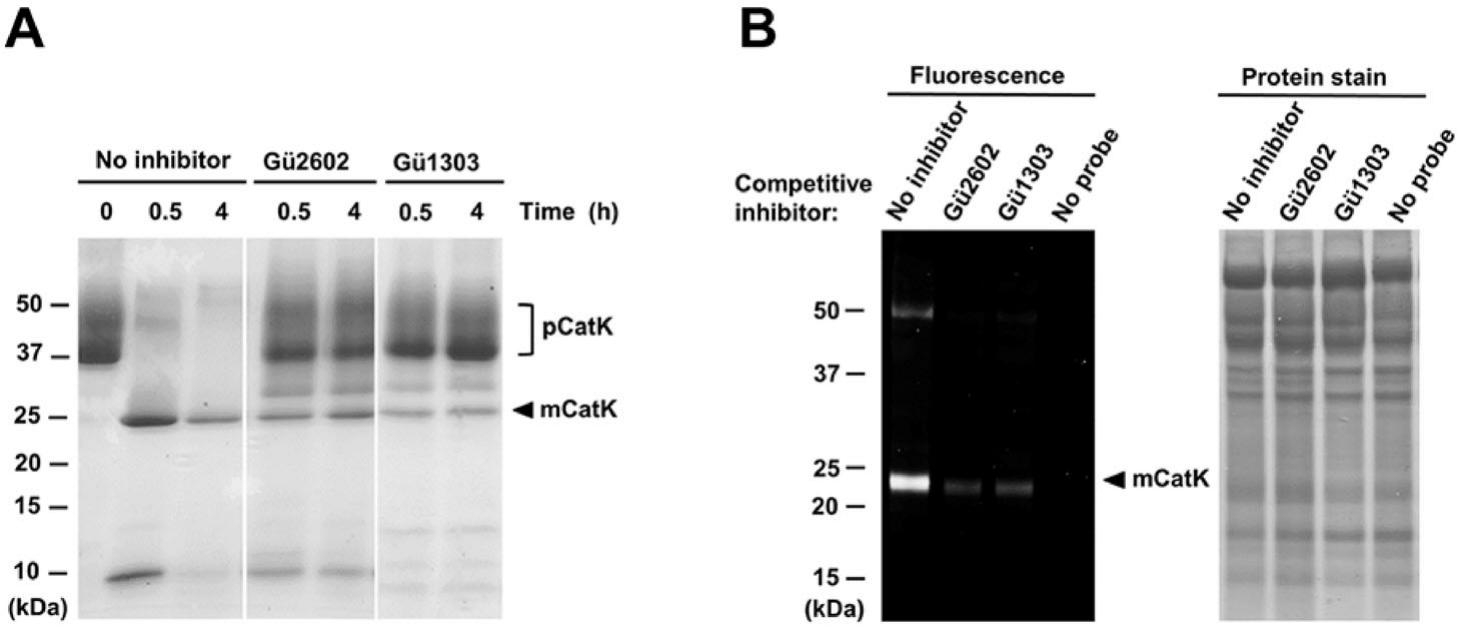
The inhibitors **Gü1303** and **Gü2602** suppress the autocatalytic activation of the cathepsin K zymogen and target cathepsin K in cells. (A) The zymogen of cathepsin K (pCatK) was incubated in the presence and absence of inhibitor (10 µM) at pH 4.0, and the generation of mature cathepsin K (mCatK) was analysed at the indicated times. The reaction mixture was resolved by SDS-PAGE and visualised by protein staining. The positions of pCatK and mCatK are indicated; note mass heterogeneity of pCatK due to glycosylation^61^. (B) The U-2 OS cells were pre-treated with inhibitor (1 µM) for 3 h, followed by 24 h incubation with a fluorescent activity-based probe specific for cathepsin K^40^ (1 μM); quenching of the labelling reaction by competitive inhibition was analysed. Cell lysates were resolved by SDS-PAGE and visualised by fluorescence imaging (left) and protein staining (right). The position of mCatK is indicated. In control experiments, the probe or inhibitor was omitted.

The mature enzyme mCatK is generated during the acidic autocatalytic processing of an inactive precursor, the cathepsin K zymogen (pCatK), and this activation process is associated with the proteolytic removal of the propeptide domain from pCatK^24–26^. We examined the effect of **Gü1303** and **Gü2602** on the autoactivation of recombinant pCatK induced by acidic pH using an *in vitro* assay with SDS-PAGE visualisation of the processing forms pCatK and mCatK (bands of ca 37 kDa and ca 24 kDa, respectively) (Figure 3(A)). Inhibitors were tested under conditions providing full conversion to mCatK and the cleaved propeptide (a band of ca 10 kDa). Both inhibitors substantially suppressed the autoactivation processing of pCatK that resulted in approximately a 10–15% conversion compared to uninhibited conditions. Also, both inhibitors were able to suppress the autodegradation of the generated mCatK observed under prolonged incubation (compare 0.5 and 4 h experiments, Figure 3(A)).

Finally, we investigated the interaction of the inhibitors with CatK using a cell-based assay with the human osteosarcoma cell line U-2 OS, which has an enhanced expression level of CatK^62^. For CatK imaging, we used a fluorescent activity-based probe that binds specifically and irreversibly to the active site of CatK^40^. Competition of the probe and inhibitor was monitored by SDS-PAGE and in-gel fluorescence. As shown in Figure 3(B), both reversible inhibitors **Gü1303** and **Gü2602** strongly diminished the mCatK labelling, demonstrating that these cyanohydrazides are cell-permeable compounds that effectively interact with the active form of CatK in the lysosomal/endosomal system.

In conclusion, the cyanohydrazide inhibitors were demonstrated to inhibit mCatK with subnanomolar potency and different binding kinetics, to suppress the generation of mCatK from its zymogen, and to effectively target active mCatK in the cell context.

### 3.2. Crystallography of complexes of two cyanohydrazide inhibitors with mature cathepsin K and its zymogen-like activation intermediate

**Figure 4.**
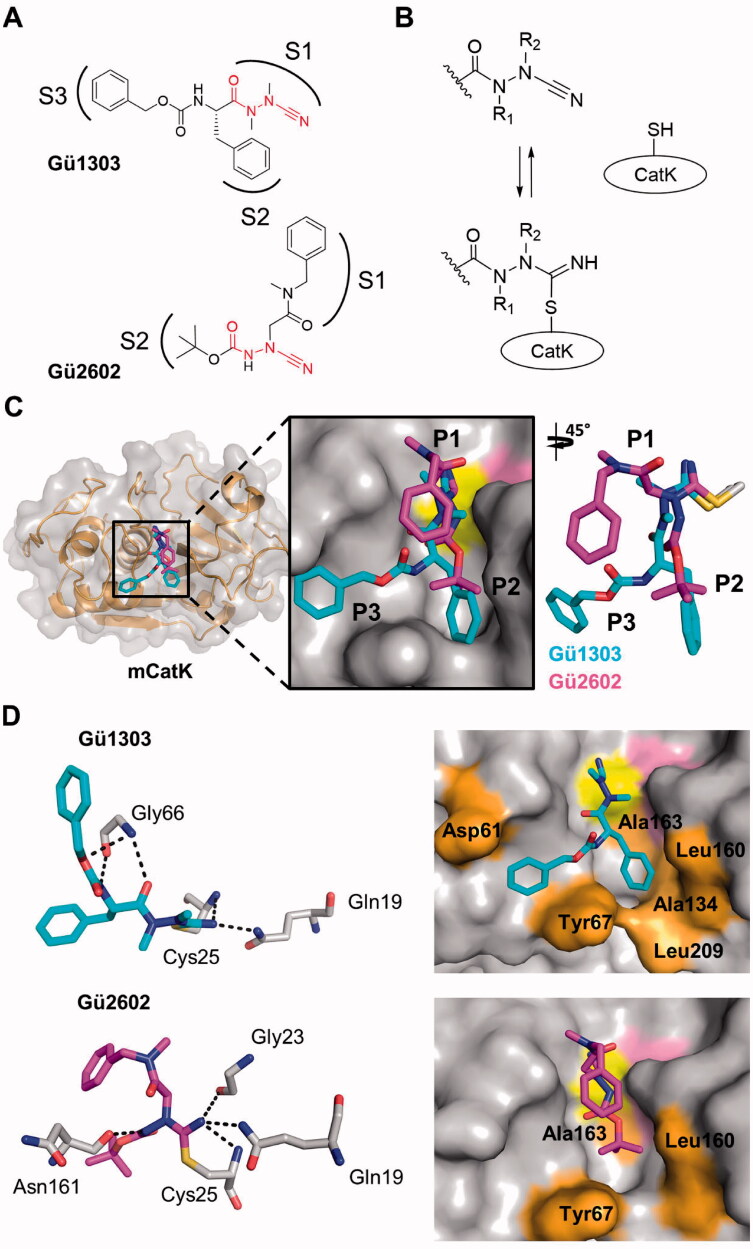
Binding mode of the cyanohydrazide inhibitors **Gü1303** and **Gü2602** in the active site of mature cathepsin K. (A) Chemical structure of the azadipeptide nitrile inhibitor **Gü1303** and 3-cyano-3-aza-β-amino acid inhibitor **Gü2602**; the binding subsites (S) are marked, and the cyanohydrazide warheads are in red. (B) Reactive warheads form a covalent reversible bond with the thiol of the catalytic cysteine residue of the enzyme; R_1_ and R_2_ are substituents on the N atoms of the warheads. (C) The zoomed-in view of the mCatK active site shows a superposition of the inhibitors bound to the S1 to S3 subsites (corresponding inhibitor positions P1 to P3 are indicated). mCatK is displayed in surface representation (grey); highlighted are the catalytic residues Cys25 (yellow) and His162 (pink). Inhibitors are shown in stick representation with carbon atoms in cyan for **Gü1303** and magenta for **Gü2602**; heteroatoms have standard colour coding (O, red; N, blue; S, yellow). (D) Interaction of the inhibitors with active site residues of mCatK. Left panels: the hydrogen bond network formed between inhibitors and mCatK residues (dashed black lines). Inhibitors are coloured as in (C), and interacting enzyme residues are in grey; the side chain of the covalently linked catalytic cysteine residue Cys25 is depicted. Right panels: the surface representation of the mCatK active site shows enzyme residues (highlighted in orange) forming nonpolar interactions with the inhibitors (in stick representation); both inhibitors are in the same orientation.

**Figure 5.**
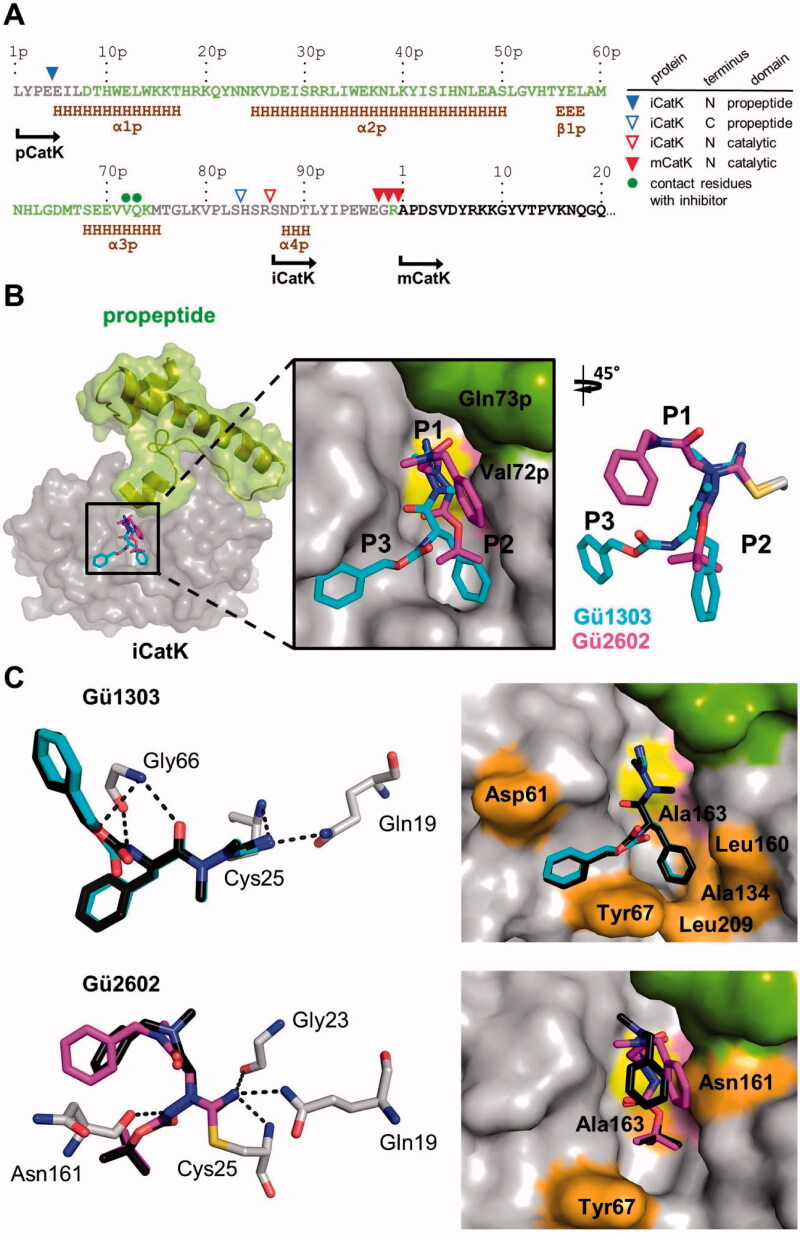
Binding mode of the cyanohydrazide inhibitors **Gü1303** and **Gü2602** in the active site of the activation intermediate of cathepsin K (iCatK). (A) The amino acid sequence of the full-length propeptide domain and the N-terminus of mature CatK (mCatK) are shown with secondary structure elements (H, α-helix; E, β-strand) (data absent in the iCatK structure are derived from the intact zymogen pCatK; PDB entry: 1BY8). Propeptide residues present or absent in the final crystallographic models of iCatK are in green or grey, respectively, residues of the catalytic domain are in black. The triangles above the sequence line indicate the N- and C-termini of the residual propeptid domain of iCatK, the N-termini of iCatK and mCatK catalytic domains as determined by the Edman sequencing and mass spectrometry (see the inset legend). The green dots show two residues of the propeptid of iCatK that form contacts with **Gü2602**. The position of the N-termini is indicated for pCatK and catalytic domains of iCatK/mCatK. (B) The zoomed-in view of the iCatK active site shows a superposition of **Gü1303** and **Gü2602** bound to the S1 to S3 subsites (corresponding inhibitor positions P1 to P3 are indicated). iCatK is displayed in surface representation, the catalytic domain is highlighted in grey, the residual propeptide domain in green, and the catalytic residues Cys25 and His162 in yellow and pink, respectively. Inhibitors are shown in stick representation with carbon atoms in cyan for **Gü1303** and magenta for **Gü2602**; heteroatoms have a standard colour coding (O, red; N, blue; S, yellow). (C) Interaction of the inhibitors with the iCatK active site residues. Left panels: the hydrogen bond network formed between inhibitors and iCatK residues with the (dashed black lines). Inhibitors are coloured as in (B), and interacting enzyme residues are in grey; the side chain of the covalently linked catalytic cysteine residue Cys25 is depicted. Superimposed (black) are the same inhibitors from the structures of their complexes with mCatK (Figure 4). Right panels: the surface representation of the iCatK active site shows enzyme residues forming nonpolar interactions (highlighted in orange) with the inhibitors (in stick representation); both inhibitors are in the same orientation. The propeptide domain residues are highlighted in green.

Human mature cathepsin K (mCatK) and a zymogen-like activation intermediate of cathepsin K (iCatK) were crystallised in complexes with two cyanohydrazide inhibitors, the azadipeptide nitrile **Gü1303** and the 3-cyano-3-aza-β-amino acid **Gü2602** (Figures 4 and 5). The crystal structures of mCatK complexes with **Gü1303** and **Gü2602** were determined by molecular replacement using the structure of uncomplexed mCatK as a template. Both complexes crystallised in the orthorhombic space group *P*2_1_2_1_2 with one molecule in the asymmetric unit (Table S1). The structure of the mCatK-**Gü1303** complex was refined using data to resolution 1.55 Å, and to 2.00 Å for mCatK-**Gü2602**. The final crystallographic models contained mCatK residues Ala1 to Met215; additional N-terminal residues derived from the propeptide, Gly-Arg (98p–99p, propeptide numbering), were visible in the mCatK-**Gü1303** structure (Figure 5). A comparison of both mCatK complexes did not reveal any significant differences in protein structure (a backbone r.m.s.d. of 0.51 Å, a value within the range observed for different crystal structures of identical proteins).

iCatK was produced by limited autocatalytic processing of the zymogen pCatK at acidic pH. This resulted in fragmentation of the propeptide with cleavage sites identified after Glu4p, Ser83p, and Arg86p residues by Edman sequencing and mass spectrometry peptide mapping analysis (Figure 5(A)). The obtained iCatK comprised two non-covalently bound domains (chains), namely the residual propeptide domain (Glu5p to Ser83p) and the main domain (Ser87p to Met215), including the C-terminal part of the propeptide and mCatK. The structure of the iCatK-**Gü2602** complex was solved by a combination of molecular replacement for the main domain (see previous paragraph) and automated model building by fragment-fitting technique for the residual propeptide. The structure of the iCatK-**Gü1303** complex was solved by molecular replacement based on the iCatK-**Gü2602** structure. Both iCatK complexes crystallised in the tetragonal space group *P*4_3_2_1_2 containing one molecule in the asymmetric unit (Table S1). The final crystallographic model of the iCatK-**Gü1303** complex contained propeptide residues Asp8p to Gln73p and main domain residues Pro2 to Met215. The model of the iCatK-**Gü2602** complex contained residues Asp8p to Lys74p and Arg99p to Met215. The structures were refined using data to resolution 1.90 Å for iCatK-**Gü1303** and 1.88 Å for iCatK-**Gü2602**. A comparison of iCatK complexes did not reveal any significant differences in protein structure (a backbone r.m.s.d. of 0.28 Å).

The residual propeptide domain of iCatK is folded in a similar manner to the intact propeptide in the structure of the zymogen pCatK (PDB: 1BY8) and bound at the same position (a backbone r.m.s.d. of 0.81 and 0.48 Å for the propeptide domains and catalytic domains, respectively). However, we observed a slightly different orientation of the α2p helix in iCatK complexes, which is rotated by approximately 10° compared to pCatK. The propeptide segment that blocks the active site in pCatK (downstream of the α3p helix, Figure 5) is proteolytically removed or flexible in iCatK, and therefore the active site cleft of iCatK becomes accessible for inhibitors. However, the residual propeptide domain partially occludes the primed region of the active site, in particular the S1’ subsite of iCatK is occupied.

### 3.3. Interaction of cyanohydrazide inhibitors with the active site of cathepsin K

#### 3.3.1. Binding mode of the azadipeptide nitrile Gü1303 to mature and zymogen-like cathepsin K

The active site cleft of mCatK contains the catalytic triad residues Cys25, His162, and Asn182. **Gü1303** is bound in a substrate-like orientation, and its P1 to P3 residues occupy the S1 to S3 subsites of mCatK (Figure 4(A,C)). The cyanohydrazide warhead reacts with the thiol group of the catalytic Cys25, forming a covalent isothiosemicarbazide adduct through the connection to the C-atom of the nitrile moiety (Figure 4(B)). Azadipeptides such as **Gü1303** are atropochiral molecules due to the restricted rotation around the methylated N–N axis, and, in the unbound state, they preferentially adopt the *E*-configuration of the respective CO–NMe bond^63^^,^^64^ (Figure 4(B)). However, a *Z*-configuration at the CO–NMe bond was observed in **Gü1303** bound to mCatK, suggesting an *E*- to *Z-*conformational change in **Gü1303** upon binding to the enzyme, most likely due to a "configurational selection" that we recently reported for an azadipeptide nitrile inhibitor of the protease SmCB1^32^.

Interactions between **Gü1303** and the mCatK active site are presented in Figure 4(D) (for details see Table S2). The inhibitor forms a network of hydrogen bonds with the active site residues. The nitrogen atom of the imidate moiety, derived from the warhead nitrile group, is stabilised by two hydrogen bonds to the backbone amide of the catalytic Cys25 and the side chain amide of Gln19 (Figure 4(D)**)**. An analogous interaction pattern was observed for the warhead of an azadipeptide nitrile inhibitor reacted with the protease SmCB1 (PDB: 6YI7)^32^. The NH of Gly66 acts as a bifurcated hydrogen bond donor for the carbonyl oxygen of the P2 phenylalanine and the noncarbonyl carbamate oxygen in the P3 position of **Gü1303.** An additional hydrogen bond is formed between the Gly66 oxygen and the carbamate NH of **Gü1303.** A similar network of Gly66-mediated hydrogen bonds was also reported for a Boc-protected precursor of a reactive activity-based probe for mCatK (PDB: 7NXL)^40^. Nonpolar interactions of **Gü1303** with mCatK are depicted in Figure 4(D). They are absent in the S1 subsite, although the warhead containing the P1 azaalanine residue forms a number of contacts (Supporting information Table S2). At the P2 position, the phenylalanine residue makes nonpolar interactions with Tyr67, Ala134, Leu160, Ala163, and Leu209. The P3 benzyloxycarbonyl capping group forms nonpolar interactions with Asp61 and Tyr67, and the phenyl moiety of this group is stabilised by a T-shaped π-π stacking interaction to the 4-hydroxyphenyl group of Tyr67.

The binding mode of **Gü1303** in the active site of iCatK is analogous to that in mCatK, and there is no interaction of the inhibitor with the residual propeptide domain that blocks a part of the primed region of the iCatK active site (Figure 5(B)). High conformational similarity of the inhibitor is indicated by a r.m.s.d. of 0.19 Å, with a certain change in the position of the P3 carbonyl oxygen. Also, **Gü1303** forms the same network of hydrogen bonds and nonpolar interactions in the S1 to S3 subsites of iCatK and mCatK (Figure 5(C), Table S2). However, no π-π stacking interaction with Tyr67 residue was observed, due to a slightly different orientation of the benzyloxycarbonyl capping group of **Gü1303**.

#### 3.3.2. Binding mode of the 3-cyano-3-aza-β-amino acid Gü2602 to mature and zymogen-like cathepsin K

**Gü2602** binds to the active site of mCatK in a substrate-like orientation, and the major conformation of the inhibitor occupies the S1 and S2 subsites (Figure 4(A,C)). The cyanohydrazide warhead positioned centrally in the inhibitor molecule forms a covalent isothiosemicarbazide adduct with a thiol group of the catalytic Cys25 (Figure 4(B)). There is a shift in the inhibitor backbone when comparing **Gü2602** and **Gü1303** that might be the result of nitrogen methylation in the warhead (CO–NH vs. CO–NMe in **Gü2602** and **Gü1303**, respectively) (Figure 4(C)). The CO–NH bond of **Gü2602** adopts the *Z*-configuration, similar to what has been observed for the CO–NMe bond of the enzyme bound inhibitor **Gü1303**.

The nitrogen atom of the imidate moiety of **Gü2602** is strongly stabilised by three hydrogen bonds to the backbone amide of the catalytic Cys25, carbonyl group of Gly23, and the side chain amide of Gln19 (Figure 4(D)). In contrast to **Gü1303**, there is no hydrogen bonding between **Gü2602** and the Gly66 residue. However, a new hydrogen bond is formed between the backbone oxygen of Asn161 and the amide NH of **Gü2602**. Analysis of the inhibitor-mCatK complexes available in the PDB shows that peptidomimetic inhibitors of mCatK frequently establish hydrogen bonding with Asn161 as well as Gly66 as important interaction determinants. The P1 and P2 residues of **Gü2602** are located in the non-primed subsites of the mCatK active site as follows (Figure 4(A,C)). The *N*-benzyl-*N*-methylacetamide substructure at P1 occupies the S1 subsite, making contacts with Gly23, Gly64, and Gly65 residues. Its terminal benzyl moiety is oriented out of the S1 subsite and towards the S2 pocket. The P2 Boc-capping group resides in the S2 subsite of mCatK and makes nonpolar interactions with Tyr67, Leu160, and Ala163 (Figure 4(D), Table S2).

In general, **Gü2602** binds to the S1 and S2 subsites of iCatK in a manner that is similar to what has been shown for the **Gü2602**-mCatK complex, with a r.m.s.d. of 1.23 Å; this value is substantially increased compared to **Gü1303** in its complexes, with a r.m.s.d. of 0.19 Å (Figure 5(B)). The major conformational change, however, is in the benzyl group of the P1 *N*-benzyl-*N*-methylacetamide substructure. It is rotated towards the residual propeptide domain of iCatK and forms new contacts with its Val72p and Gln73p residues (Figure 5(A,B), Table S2). The terminal Boc group accommodates the S2 pocket of iCatK analogously as in mCatK. The network of hydrogen bonds is identical, and the pattern of nonpolar interactions (Tyr67, Asn161, and Ala163 in iCatK) is similar for both **Gü2602** complexes (Figure 5(C)).

### 3.4. Conformational flexibility of cyanohydrazide inhibitors in the cathepsin K active site

**Figure 6.**
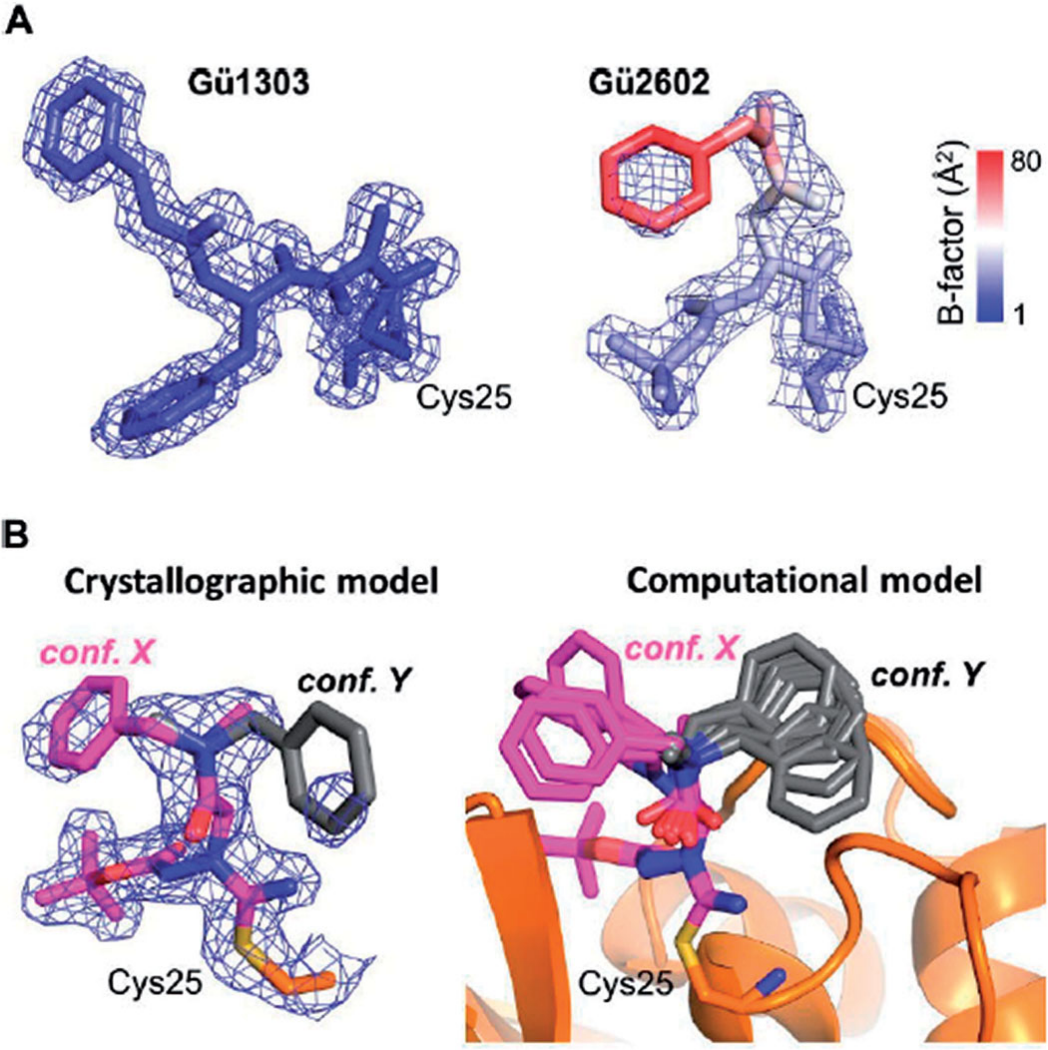
Analysis of conformational flexibility of **Gü1303** and **Gü2602** inhibitors in the active site of mature cathepsin K. (A) The inhibitors and the side chain of the covalently linked catalytic cysteine residue Cys25 are shown in stick representation. Their 2*F*_o_-*F*_c_ electron density maps are contoured at 1 σ and 1.5 σ for **Gü1303** and **Gü2602**, respectively. Structures are coloured according to atomic B-factor values, from blue (low) to red (high). The highest B-factors indicating flexibility are observed for the benzyl moiety of **Gü2602**. (B) Conformational flexibility of the benzyl moiety of **Gü2602**. Left panel: Crystallographic model of **Gü2602** with the major observed conformation of the benzyl moiety in magenta (conf. X located in the non-primed area) and a predicted alternative minor conformation in grey (conf. Y located in the primed area). The side chain of the covalently linked catalytic Cys25 is in orange; heteroatoms have a standard colour coding (O, red; N, blue; S, yellow). The 2*F*_o_-*F*_c_ electron density maps are contoured at 1 σ. Right panel: Conformations of the benzyl moiety were searched by the molecular dynamics/quenching (MD/Q) technique and optimised by the semiempirical quantum mechanical (SQM) method. The lowest-energy conformations up to relative Gibbs “free” energy of 5 kcal/mol are shown for the general orientations X and Y (as defined by crystallography in left panel).

The crystallographic electron density maps used for modelling **Gü1303** and **Gü2602** in the active site of mCatK were of high quality except for a weak electron density signal of the benzyl group of **Gü2602** (Figure 6(A)). This prompted us to analyse the B-factor distribution in both inhibitors. The B-factor values were generally in the low range, but increased values were only found for the P1 *N*-benzyl-*N*-methylacetamide part of **Gü2602,** with the highest value for its benzyl group (Figure 6(A)). An analogous B-factor pattern was also observed for inhibitors in iCatK complexes (Figure S1). This finding indicated an increased flexibility and dynamic disorder of the major crystallographic conformation of the benzyl group of **Gü2602**, which is located in the non-primed part of the active site and oriented out of the S1 subsite (Figure 5(C)).

A detailed inspection of electron density maps in the active site of the mCatK-**Gü2602** complex provided a further weak electron density signal of poor quality in the primed region of the mCatK active site that can be assigned to **Gü2602**. Based on this signal, we attempted to model an alternative conformation of the benzyl group of **Gü2602**, which is oriented towards the S2’ subsite as presented in Figure 6(B) (see conformation Y). For this purpose, we employed molecular modelling to examine conformational space of the flexible *N*-benzyl-*N*-methylacetamide substructure of **Gü2602.** The molecular dynamics/quenching (MD/Q) technique was utilised to generate accessible conformations of this segment in the active site, and semiempirical quantum mechanical optimisation yielded a set of conformations with two general orientations into the non-primed and primed areas (the orientations are marked X and Y, respectively, in Figure 6(B)).

In conclusion, we demonstrated that **Gü2602** is more flexible in the enzyme active site in contrast to the rigid ligand **Gü1303**. The former contains a highly flexible benzyl moiety that is capable of adopting to two different types of conformations that reside in the non-primed part or primed part of the active site.

## 4. Discussion and conclusions

Cathepsin K (CatK) is a target for the treatment of osteoporosis, arthritis, and bone metastasis, and its potent and selective inhibitors are being intensively pursued as chemotherapeutics^6^^,^^7^^,^^24^. In this study, we investigated peptidomimetic inhibitors with a cyanohydrazide warhead, a class of highly efficient inhibitors of CatK that have been recently discovered^30^^,^^31^^,^^39^, yet for which the interaction mechanism at the atomic level with CatK is unknown. For two representative cyanohydrazide compounds, **Gü1303** and **Gü2602**, we present crystallographic analysis of the binding mode to mature enzyme mCatK and its zymogen-like activation intermediate iCatK, as well as functional analysis *in vitro* and in cells.

The crystal structures of the complexes of **Gü1303** or **Gü2602** with mCatK showed that the inhibitors are bound in a substrate-like orientation, and their cyanohydrazide warhead reacted with a thiol group of the catalytic Cys25, forming a covalent isothiosemicarbazide adduct. The CO–NMe and CO–NH bond (in **Gü1303** and **Gü2602**, respectively) of the warhead adopted a *Z*-configuration in the mCatK active site. This is in line with our recent analysis of another cyanohydrazide inhibitor (with the azadipeptide nitrile scaffold) in the active site of a cysteine protease^32^ which also demonstrated that the warhead with the methylated N–N axis provides atropochirality, and the *E*-configuration of the unbound inhibitor is transformed to a Z-configuration upon binding^32^. Therefore, such an *E*- to *Z*-conformational change in the course of its interaction with CatK is proposed for **Gü1303**, containing the CO–NMe–NMe portion.

The cyanohydrazide warhead is differently positioned in the inhibitor scaffold of the azadipeptide nitrile **Gü1303** and 3-cyano-3-aza-β-amino acid **Gü2602**. This is reflected in the distribution of the binding subsites that are targeted by the inhibitors. **Gü1303** occupies the non-primed subsites S1 to S3, and B-factor distribution showed that **Gü1303** is rigid in the mCatK active site. In contrast, **Gü2602** primarily occupies the S2 subsite, and the *N*-benzyl-*N*-methylacetamide part (especially its benzyl moiety) is highly flexible and has two alternative types of conformations that reside in the non-primed part (the S1 subsite) or primed part (the S2’ subsite) of the active site. The non-primed orientation is the major one observed in the crystal structure, where it might be stabilised by additional contacts, including crystal packing contacts (with a symmetry-related protein molecule) and intramolecular contacts. The primed orientation was clearly demonstrated by molecular modelling using MD/Q and SQM techniques and is further supported by the recent identification of a benzyl group oriented towards the S2’ pocket in the activity-based probe for CatK^40^. The computational approach showed a set of conformations for both types of orientations, further highlighting the high flexibility of this part of **Gü2602** in the enzyme active site.

We hypothesise that the conformational flexibility of the complexed ligand at the boundary of the non-primed and primed subsites provides an entropic advantage contributing to the extraordinary potency of **Gü2602** in the low picomolar range. It has been shown that the loss of configurational entropy upon ligand binding contributes significantly unfavourably to the binding free energy^65^; this entropy loss is decreased for **Gü2602** making its binding more favourable. An analysis of active site interactions formed by **Gü2602** also identified an important role of hydrogen bonding between the amide NH of the warhead and Asn161 of the S2 subsite, an interaction that is absent in **Gü1303**. This is supported by analogues of **Gü2602** with methylated NH that exhibited a dramatic decrease in inhibitory potency by 3 orders of magnitude^39^^,^^40^.

The differences in the binding mode between **Gü2602** and **Gü1303** are also reflected in their kinetic behaviour. The slow-binding of **Gü1303** is attributed to a conversion from an *E*- to *Z*-configuration upon binding that was recently described as the kinetic controlling step for a prototype azadipeptide nitrile with an atropochiral warhead^32^. In contrast, the 3-cyano-3-aza-β-amino acid scaffold of **Gü2602** bears a non-atropochiral warhead, as demonstrated by NMR studies showing the absence of diastereotopic methylene protons^39^. Hence, the configuration conversion step does not occur, as is reflected by the fast-binding of **Gü2602** that resembles analogous kinetics and a reaction mechanism described for carbanitrile inhibitors^32^.

In addition to mCatK-inhibitor complexes, we also structurally characterised the complexes of **Gü2602** and **Gü1303** with the activation intermediate iCatK produced during autocatalytic processing of the inactive zymogen to mature enzyme that is associated with the removal of the propeptide. The iCatK structure is described here for the first time. It contains the main catalytic domain, which is non-covalently bound to the residual propeptide domain. Unlike the zymogen structure^27^^,^^28^, the residual propeptide (residues 4p to 83p) of iCatK only partially occluded the active site cleft (mainly the S1’ subsite), and thus the catalytic centre is accessible for ligands. This contrasts to the previously investigated activation intermediate of the cathepsin SmCB1, in which the catalytic centre is still blocked by the residual propeptide^41^.

iCatK was demonstrated to bind **Gü2602** and **Gü1303** in an analogous manner to that observed in the mCatK complexes. This indicated that the iCatK active site is competent to bind inhibitory ligands in the available subsites and can be regulated by them. This conclusion is supported by the results on testing **Gü2602** and **Gü1303** in an *in vitro* autoactivation assay with the zymogen pCatK. It showed that both inhibitors strongly suppress the formation of mCatK from the zymogen that proceeds as a bimolecular processing reaction catalysed by functional forms of iCatK/mCatK. Furthermore, we provided evidence that **Gü2602** and **Gü1303** are capable of effectively targeting mCatK as well as the mCatK-generating pathway in the pathophysiologically relevant context of osteosarcoma cells.

In conclusion, our work provides the first crystallographic, computational chemical, and functional insights into the binding mode of the cyanohydrazide inhibitors to the CatK target and will facilitate a further rational design of therapeutics against CatK-mediated pathologies. 

## Supplementary Material

Supplemental Material

## Data Availability

Atomic coordinates and experimental structure factors have been deposited in the Protein Data Bank with accession codes 7QBL, 7QBM, 7QBN, and 7QBO.
