# Supplementary material for: Highly potent inhibitors of cathepsin K with a differently positioned cyanohydrazide warhead: structural analysis of binding mode to mature and zymogen-like enzymes
Source: J Enzyme Inhib Med Chem. 2022 Feb 11;37(1):515–26. doi: 10.1080/14756366.2021.2024527 (PMC8843313; doi:10.1080/14756366.2021.2024527)
Supplement: Supplemental Material [file IENZ_A_2024527_SM4071.pdf]

Supplementary materials

Highly potent inhibitors of cathepsin K with a differently positioned cyanohydrazide warhead: structural analysis of binding mode to mature and zymogen-like enzyme

Jakub Benýšek[a,b], Michal Buša[a,c], Petra Rubešová[a], Jindřich Fanfrlík[a], Martin Lepšík[a], Jiří Brynda[a], Zuzana Matoušková[a], Ulrike Bartz[d], Martin Horn[a], Michael Gütschow[e], Michael Mareš*[a]

[a]Institute of Organic Chemistry and Biochemistry of the Czech Academy of Sciences, Flemingovo n. 2, 16610 Prague, Czech Republic

[b]First Faculty of Medicine, Charles University, Kateřinská 1660/32, 12108 Prague, Czech Republic

[c]Department of Biochemistry, Faculty of Science, Charles University, Hlavova 2030/8, 12800 Prague, Czech Republic

[d]Department of Natural Sciences, University of Applied Sciences Bonn-Rhein-Sieg, von-Liebig-Strasse 20, D-53359 Rheinbach, Germany

[e]Pharmaceutical Institute, Pharmaceutical & Medicinal Chemistry, University of Bonn, An der Immenburg 4, 53121 Bonn, Germany

*Corresponding author: mares@uochb.cas.cz

Table S1. X-ray data collection and refinement statistics

CatK-inhibitor complex[a]	mCatK-Gü2602	mCatK-Gü1303	iCatK-Gü2602	iCatK-Gü1303
Data Collection Statistics				
wavelength (Å)	1.542	1.542	0.918	0.918
temperature (K)	100	100	100	100
space group	$P2_12_12$	$P2_12_12$	$P4_32_12$	$P4_32_12$
a, b, c (Å)	70.63, 78.52, 31.02	74.94, 76.12, 32.85	103.28, 103.28, 55.46	103.21, 103.21, 54.99
α, β, γ (deg)	90.00, 90.00, 90.00	90.00, 90.00, 90.00	90.00, 90.00 ,90.00	90.00, 90.00, 90.00
resolution (Å)	50.0–2.00 (2.12–2.00)	50.0–1.55 (1.64–1.55)	50.0–1.88 (1.92–1.88)	50.0–1.90 (2.02–1.90)
number of unique reflections	11 829 (1880)	23 646 (1843)	24 933 (1541)	23 911 (3763)
redundancy	4.1 (4.0)	6.5 (4.7)	11.1 (4.8)	7.9 (7.5)
completeness (%)	96.5 (97.8)	83.4 (41.0)	99.7 (96.4)	99.8 (99.3)
R_{merge}[b] (%)	7.8 (59.2)	8.5 (38.7)	18.7 (214.1)	11.1 (63.0)
average I/σ (I)	12.4 (2.5)	19.4 (4.4)	7.9 (0.7)	13.3 (2.9)
$CC_{1/2}$[c] (%)	99.8 (88.9)	99.8 (89.1)	97.2 (31.5)	99.8 (85.3)
Wilson B ($Å^2$)	35.06	15.94	28.40	27.48
Refinement Statistics				
resolution range (Å)	28.90–2.0 (2.05–2.0)	37.50–1.55 (1.59–1.55)	48.91–1.88 (1.93–1.88)	46.20–1.90 (1.95–1.90)
number of reflections in working set	11 344 (836)	22 487 (439)	23 673 (1662)	22 715 (1633)
number of reflections in test set	598 (44)	1184 (23)	1213 (84)	1196 (86)
R value[d] (%)	22.1 (35.5)	14.4 (23.8)	19.0 (37.4)	16.4 (25.5)
R_{free} value[e] (%)	28.6 (34.5)	16.6 (26.0)	23.4 (37.5)	212 (29.1)
number of molecules in AU[f]	1	1	1	1
number of atoms in AU[g] protein/inhibitor/solvent	1657/23/47	1705/27/302	2226/23/235	2226/27/293
average ADP[g] for protein/inhibitor/solvent ($Å^2$)	38.0/43.2/33.0	12.9/14.8/24.3	36.9/49.2/40.5	24.0/29.7/33.2
RMSD bond length (Å)	0.014	0.015	0.014	0.014
RMSD bond angle (deg)	1.91	1.77	1.74	1.68
Ramachandran plot statistics[h]				
favoured regions (%)	95.8	97.7	95.3	97.9
allowed regions (%)	4.2	2.3	4.7	2.2
PDB code	7QBL	7QBN	7QBM	7QBO

[a]Numbers in parentheses refer to the highest-resolution shell. [b]$R_{merge}=100\sum_{hkl}\sum_i|I_i(hkl) - \langle I(hkl)\rangle|/\sum_{hkl}\sum_i I_i(hkl)$, where $I_i(hkl)$ is an individual intensity of the ith observation of reflection *hkl* and ⟨I(*hkl*)⟩ is the average intensity of reflection *hkl* with summation over all data. [c]$CC_{1/2}$ is the percentage of correlation between intensities from random half-datasets.[1] [d]R-value = $||F_o| - |F_c||/|F_o|$, where F_o and F_c are the observed and calculated structure factors, respectively. [e]R_{free} is equivalent to R value but is calculated for up to 5% of the reflections chosen at random and omitted from the refinement process.[2] [f]AU, asymmetric unit. [g]ADP, atomic displacement parameter, formally B-factor. [h]As determined by Molprobity.[3]

Table S2. List of contacts formed between the inhibitors **Gü1303** and **Gü2602** and mature cathepsin K (mCatK) or the activation intermediate of cathepsin K (iCatK). Analysis of protein-inhibitor contacts between the mCatK/iCatK active site and inhibitors was performed using program CONTACT.[4] The distance cutoffs were set to 4.2 Å for all contacts and 3.3 Å for hydrogen bonds. The enzyme residues interacting in the individual inhibitor positions (P3 to P1) are specified. For each enzyme residue, total number of contacts (C) are listed, including hydrogen bonds (Hb) (residues forming hydrogen bonds are in bold).

Subsite	mCatK-Gü2602			mCatK-Gü1303			iCatK-Gü2602			iCatK-Gü1303		
	Residue	C	Hb	Residue	C	Hb	Residue	C	Hb	Residue	C	Hb
P1							Val72p	10				
							Gln73p	2				
	**Gln19**	2	**1**	**Gln19**	3	**1**	**Gln19**	2	**1**	**Gln19**	3	**1**
	**Gly23**	10	**1**	Gly23	10		**Gly23**	9	**1**	Gly23	10	
	Ser24	2		Ser24	3		Ser24	2		Ser24	3	
	**Cys25**	13	**1**	**Cys25**	14	**1**	**Cys25**	13	**1**	**Cys25**	14	**1**
	Trp26	2		Trp26	3		Trp26	1		Trp26	3	
	Cys63	1					Cys63	1				
	Gly64	7					Gly64	4				
	Gly65	2					Gly65	4				
	**Asn161**	10	**1**	Asn161	2		**Asn161**	6	**1**	Asn161	2	
	His162	3		His162	2		His162	3		His162	2	
P2	Cys25	1		Cys25	2		Cys25	2		Cys25	2	
	Gly65	2		Gly65	6		Gly65	2		Gly65	6	
	Gly66	5		**Gly66**	11	**2**	Gly66	5		**Gly66**	11	**2**
	Tyr67	2		Tyr67	3		Tyr67	2		Tyr67	3	
				Met68	2					Met68	2	
				Ala134	3					Ala134	3	
	Leu160	3		Leu160	4		Leu160	3		Leu160	4	
	Asn161	6					Asn161	9				
	His162	3					His162	3				
	Ala163	1		Ala163	1		Ala163	1		Ala163	1	
				Leu209	2					Leu209	2	
P3				Glu59	3					Glu59	3	
				Asn60	3					Asn60	3	
				Asp61	5					Asp61	5	
				Gly65	3					Gly65	3	
				**Gly66**	15	**1**				**Gly66**	15	**1**
				Tyr67	11					Tyr67	11	

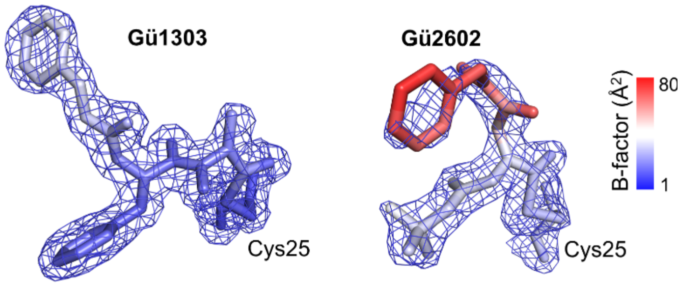

Figure S1. Analysis of conformational flexibility of the inhibitors **Gü1303** and **Gü2602** in the active site of the activation intermediate of cathepsin K. The inhibitors and the side chain of the covalently linked catalytic cysteine residue Cys25 are shown in stick representation. Their $2F_o$-F_c electron density maps are contoured at 1 σ and 1.5 σ for **Gü1303** and **Gü2602**, respectively. Structures are coloured according to atomic B-factor values, from blue (low) to red (high). The highest B-factors indicating flexibility are observed for the benzyl moiety of **Gü2602**.

References

1. Karplus, P. A.; Diederichs, K., Linking crystallographic model and data quality. *Science* **2012,** *336* (6084), 1030-1033.
2. Brünger, A. T., Free R value: a novel statistical quantity for assessing the accuracy of crystal structures. *Nature* **1992,** *355* (6359), 472-475.
3. Lovell, S. C.; Davis, I. W.; Arendall, W. B., 3rd; de Bakker, P. I.; Word, J. M.; Prisant, M. G.; Richardson, J. S.; Richardson, D. C., Structure validation by Calpha geometry: phi, psi and Cbeta deviation. *Proteins Struct Funct Bioinform* **2003,** *50* (3), 437-450.
4. Winn, M. D.; Ballard, C. C.; Cowtan, K. D.; Dodson, E. J.; Emsley, P.; Evans, P. R.; Keegan, R. M.; Krissinel, E. B.; Leslie, A. G.; McCoy, A.; McNicholas, S. J.; Murshudov, G. N.; Pannu, N. S.; Potterton, E. A.; Powell, H. R.; Read, R. J.; Vagin, A.; Wilson, K. S., Overview of the CCP4 suite and current developments. *Acta Crystallogr D Biol Crystallogr* **2011,** *67* (Pt 4), 235-242.
